# Pain and Medical Research
**For and Against Experiments on Animals.* By Stephen Paget, F.R.C.S. (Hon. Secretary Research Defence Society). With an Introduction by the Right Hon. the Earl of Cromer. (London: H. K. Lewis, 136 Gower Street. 1912. Price 3s. 6d.)


**Published:** 1912-09-14

**Authors:** 


### PAIN AND MEDICAL RESEARCH.*

Voluminous, inchoate, highly technical, the preliminary minutes of the evidence tendered before the Royal Commission on Vivisection may well appal the reader, whether he be doctor or interested layman. Even the report and memoranda, clear and succinct as they are, have an air of imponderable seriousness which does not attract. Here and there in these tomes gleams a flash of humour, conscious or unconscious, but the mass of text is unrelieved. Only one who is greatly minded to study the problems with which the reports deal will have the leisure and the energy to plod courageously through all that print, finding, very likely, at the end, that his brain is in a whirl and that he is as impotent to form definite conclusions as he was before. Mr. Paget's summary of the evidence—for this book is little more than an epitome of the various reports—will, therefore, be welcomed.

It is a fair compilation, as much impartial as it can be made by one whose sympathies are obviously with the much-maligned scientific investigator. The preface by Lord Cromer is in a sense an apology for the personal views of the late President of the Research Defence Society—an apology, that is, in the best meaning of the term, explanatory as well as valedictory. The Society owes much to Lord Cromer's warm appreciation of the good that has been done by patient research—an appreciation which is not diminished by the fullest recognition that there are, and have been, mistakes made in the interpretation of the facts drawn from such investigations.

So, also, is the evidence condensed in this book, for it reveals clearly the state of mind of at least three different classes whose opinions are at least worth some respect. There is, first, the common or garden upholder of vivisection, who bases his position on the assumption that whatever pain be inflicted on animals, such suffering is justifiable if from it and through it data are obtainable that may be of real value in increasing our knowledge of the treatment and prevention of human suffering. These investigators refuse to admit "moral" arguments. They maintain that Nature is more cruel than any laboratory enthusiast; that pain is a necessary evil, if it be an evil; and that it is no more cruel to cut out a dog's sweetbread in order to glean one or two facts that may have a very close bearing on the pathology of diabetes than it is to deprive bees of their honey. They point out, again quite justifiably, that hundreds of "vivisection experiments" are done daily, and done cruelly, by laymen. The terrier has his tail shortened—without an anæsthetic being given; the horse is castrated—without chloroform; thousands of maimed birds and beasts perish miserably every season on the moors or in the woods as the result of so-called sport. To adduce such examples, we say, is fair in the sense that the human race deliberately inflicts pain in other directions, apparently with no qualm of conscience at all. Dr. Pembrey's evidence reads admirably, for he accepts the full responsibility that the investigator, if he be honest, must take upon himself in justification for his work.

Secondly, there is a class which does not confess its responsibilities quite so frankly. Some investigators, such as Professor Starling, for instance, appear to deny that their experiments are painful; they draw a distinction between "physical and mental pain." Their position is not logical, or better canonical, but they argue that under present conditions vivisection in this country is never cruel, and they urge that it has attained important results, the benefit of which to the human race only the third class will be disposed to deny. Their evidence, it seems to us, would be all the stronger if they were better acquainted with the history of medicine than some of them appear to be. It is not right to say that the possibility of intestinal resection "was only established by repeated trials by physiologists on the lower animals" (Professor Starling), nor that "there is not a single function of the nervous system, the principle of which we know now, which is not derived from experiments on animals" (Sir Victor Horsley). Sir Victor's statement, apart from its slipshod construction, may be excused if by animals we understand man as well as the lower animals.

There is surely sufficient evidence to prove the utility of scientific experiments on animals without resorting to ambiguous statements which the student of the history of medicine cannot accept. There are several such slips, especially in the physiologists' evidence. Is it true that "anybody who would be asked now to write an article on pyæmia or blood-poisoning in a dictionary of surgery could not do it; the diseases (*sic*) are gone"?

There is a third class, mainly composed of folk who take a high moral stand and object to vivisection as a diabolical and absolutely indefensible outrage on humanity. We who know what the diphtheria ward is to-day and remember what it was twenty years ago can scarcely comprehend the state of mind of such a witness as Mr. Lewis, who said that he would not allow his own child to have antitoxin administered to save its life. Yet he, too, is logical. His premisses may seem to be intellectual absurdities, but from them he works to a conclusion which, however stupendous, is strictly fair. Admitting the immorality of a practice, is it right to accept the benefits such immorality brings, even to save suffering and disease? Some scientists, who would have all anti-vivisectionists of the stern calibre of this clergyman condemn those who, while denying the right of science to use dumb animals as the subjects of experiments, nevertheless avail themselves of the results obtained by such experiments. Sir Fletcher Moulton apparently objects, in a sentence which also needs revision, to the lady doctor who cures her patients by messy things which have been discovered by vivisection. "We are now," he says, "thanks to the theoretical (*sic*) knowledge that we have acquired of the causes of these diseases, working to help Nature along the very lines which she has from the foundation of the world herself taken in fighting these diseases." That is a statement which no manner of argument will ever make the third class accept. Between the beliefs of the logical vivisector and the anti-vivisector of the sternly logical class there is an unbridgeable gulf. On the other hand, there is a point where the second class and the majority of those who object to vivisection on the ground of its cruelty may meet. Mr. Coleridge, for example, admits the advantages to be gained from research; Dr. Snow would allow experiments on animals to try new drugs and methods. Such objectors object to cruelty, to useless and unnecessary experimentation, and to the corybantic surgery that is common enough in other countries. As an impartial summary of the evidence and of the findings of the inquiry it is welcome to those who have not the leisure to read the large reports issued by the Royal Commission.

* *For and Against Experiments on Animals.* By Stephen Paget, F.R.C.S. (Hon. Secretary Research Defence Society). With an Introduction by the Right Hon. the Earl of Cromer. (London: H. K. Lewis, 136 Gower Street. 1912. Price 3s. 6d.)

